# Audit of Current Vitamin D Testing on Bridge House Detoxification Unit

**DOI:** 10.1192/bjo.2024.534

**Published:** 2024-08-01

**Authors:** Segun Ayanda, Francis Felix, Sally Braithwaite, Annie McCloud

**Affiliations:** Kent and Medway Partnership Trust, Maidstone, United Kingdom

## Abstract

**Aims:**

The National Institute for Health and Care Excellence (NICE) recommends routine testing and replacing vitamin D in adults considered high risk of deficiency. Evidence suggests high prevalence of vitamin D deficiency among those with alcohol dependence and those with chronic liver disease regardless of etiological factors. These findings are particularly important for Bridge House Detoxification Unit, where patients with complex substance use disorder (SUD), and multiple physical and mental health co-morbidities, undergo detoxification. The purpose of this audit project was to establish current levels of vitamin D testing on Bridge House Detoxification Unit in comparison to the standard set by NICE guideline PH56, and to improve it.

**Methods:**

Data was collected retrospectively from a total of 76 patients, through 3 rounds of data collection. In each round all the patients discharged within a 2 months period were included. The Audit tool looked at whether vitamin D was tested on admission. Vitamin D level ranges were defined according to the Royal Osteoporosis Society: <25nmol/L is deficient, 25–50nmol/L inadequate, >50nmol/L is sufficient. After the first round, an intervention in the form of pre-populated blood form including vit D testing was introduced. This was to be used on the first day of admission. The second round measured improvement while the third round measured maintenance. Microsoft Excel was used to analyse data.

**Results:**

During the first round of data collection, no patient had their vitamin D tested. Following our intervention, 86.67% of our patients had their vitamin D tested suggesting significant improvement to compliance in the second round. In the third round, we were able to maintain compliance at 90%. Of the 44 patients that had their vitamin D tested after our intervention, 30 (68.18%) patients were within the deficient and inadequate thresholds, requiring vitamin D replacement.

**Conclusion:**

This audit project examined international literature and local data identifying that vitamin D is indeed low among our patient group, therefore should be regarded as a high-risk group for vitamin D deficiency. There is sufficient evidence among the international literature that people with SUD suffer through significant physical and mental health effects of low vitamin D. A simple intervention of a prepopulated blood form was able to increase our compliance and maintained this.

